# Individual differences in the influence of task-irrelevant Pavlovian cues on human behavior

**DOI:** 10.3389/fnbeh.2015.00163

**Published:** 2015-06-24

**Authors:** Sara Garofalo, Giuseppe di Pellegrino

**Affiliations:** ^1^Center for Studies and Research in Cognitive Neuroscience, Department of Psychology, University of BolognaCesena, Italy; ^2^Department of Psychiatry, University of CambridgeCambridge, UK; ^3^Behavioural and Clinical Neuroscience Institute, Department of Psychology, University of CambridgeCambridge, UK

**Keywords:** Pavlovian-to-instrumental transfer, cue-controlled behavior, Sign-Tracker, Goal-Tracker, reinforcement learning

## Abstract

Pavlovian-to-instrumental transfer (PIT) refers to the process of a Pavlovian reward-paired cue acquiring incentive motivational proprieties that drive choices. It represents a crucial phenomenon for understanding cue-controlled behavior, and it has both adaptive and maladaptive implications (i.e., drug-taking). In animals, individual differences in the degree to which such cues bias performance have been identified in two types of individuals that exhibit distinct Conditioned Responses (CR) during Pavlovian conditioning: Sign-Trackers (ST) and Goal-Trackers (GT). Using an appetitive PIT procedure with a monetary reward, the present study investigated, for the first time, the extent to which such individual differences might affect the influence of reward-paired cues in humans. In a first task, participants learned an instrumental response leading to reward; then, in a second task, a visual Pavlovian cue was associated with the same reward; finally, in a third task, PIT was tested by measuring the preference for the reward-paired instrumental response when the task-irrelevant reward-paired cue was presented, in the absence of the reward itself. In ST individuals, but not in GT individuals, reward-related cues biased behavior, resulting in an increased likelihood to perform the instrumental response independently paired with the same reward when presented with the task-irrelevant reward-paired cue, even if the reward itself was no longer available (i.e., stronger PIT effect). This finding has important implications for developing individualized treatment for maladaptive behaviors, such as addiction.

## Introduction

Goal-directed behavior can be variably influenced by external and internal factors which impact the values and priorities assigned to rewards and goals (Doya, 2008). One of the most simple and effective mechanisms for influencing choice is reinforcement learning. Reinforcement learning allows animals to connect spatially and/or temporally related events in order to predict future events. Given the complexity of the animal’s environment, learning that an arbitrary cue (e.g., a sound) is predictive of a certain goal (e.g., obtain a reward, such as food), allows the animal to learn a flexible response that facilitates achievement of the goal itself. In most cases such *cue-controlled behavior* is adaptive; for example it helps one obtain food when hungry (Perks and Clifton, 1997; Holmes et al., 2010). However, an inflexible association can lead to perseverance in the same choice even if the goal itself is no longer available, or has negative long-term consequences (Holmes et al., 2010). For example, a cue associated with drugs can induce relapse even when the drug is not voluntary sought, and a sign associated with food can induce craving in the absence of hunger, leading to compulsive over-eating (Volkow et al., 2008). These biases on voluntary choice are also implemented in marketing strategies, such as advertisements, to influence consumer behavior (Smeets and Barnes-Holmes, 2003; Bray et al., 2008; de Wit and Dickinson, 2009). Cue-controlled behaviors have been interpreted as the endpoint of an initial intentional seeking behavior (of a reward), which leads to habitual, and ultimately compulsive, conduct characterized by a loss of control over behavior (Everitt and Robbins, 2005). This interesting framework proposes that the transition from intentional volition to habit and compulsion can be explained by interactions between Pavlovian and instrumental learning processes: a reward acts as an instrumental reinforcer by enhancing actions that are able to produce it, while Pavlovian learning confers incentive salience to cues (Conditioned Stimuli or CS) closely associated with the reward (Everitt and Robbins, 2005). Such cues can elicit craving and motivation towards the associated reward, thus biasing choice. Well-known evidence of this effect can be found in the so-called Pavlovian-to-Instrumental Transfer (PIT) effect (Estes, 1943, 1948). PIT captures the ability of a Pavlovian cue (i.e., a CS associated with a reward) to increase the likelihood of an instrumental response independently paired with the same (specific-PIT), or a similar (general-PIT), reward (Rescorla and Solomon, 1967; de Wit and Dickinson, 2009; Holmes et al., 2010). This effect emerges without any formal association between Pavlovian and instrumental contingencies, and even when the reward itself is no longer available (Talmi et al., 2008). PIT has been mainly studied in non-human animals (Rescorla and Solomon, 1967; Lovibond, 1981; Colwill and Rescorla, 1988; Balleine, 1994; Rescorla, 1994a, 1997, 2000; Delamater, 1995, 1996; Holland et al., 2002; Corbit and Balleine, 2003; Holland and Gallagher, 2003; Holland, 2004; Delamater and Holland, 2008; for review, see Dickinson and Balleine, 1994, 2002; Holmes et al., 2010), but some recent studies have also reported this effect in humans (Paredes-Olay et al., 2002; Hogarth et al., 2007, 2010, 2013a,b; Bray et al., 2008; Allman et al., 2010; Nadler et al., 2011; Prévost et al., 2012; Lovibond and Colagiuri, 2013).

An important, but still neglected, aspect in the human literature about PIT concerns individual differences. In the animal literature, the extent to which a Pavlovian cue becomes attractive and exerts a biasing effect varies between individuals. In particular, Sign-Trackers (ST) and Goal-Trackers (GT) have been shown to have different learning styles, consisting of a tendency to attribute more or less incentive salience to Pavlovian reward-associated cues. In a typical Pavlovian conditioning paradigm, a CS (e.g., lever presentation) is paired with a reward (e.g., food pellet), which is delivered in a different spatial position. In such a situation, two different Conditioned Responses (CR; i.e., learned responses to a previously neutral stimulus) might be expressed. Some animals approach and engage the CS (the Sign) itself and, only after its termination, reach the location of reward delivery; other animals, upon CS presentation, immediately engage the location of reward delivery (the Goal), even if it is not yet available. The first CR has been categorized as Sign-Tracking behavior, while the second CR has been categorized as Goal-Tracking behavior. ST and GT can be conceived of as different learning styles, expressed through a specific CR during Pavlovian learning. ST behavior is thought to arise from the attribution of incentive salience to Pavlovian reward-paired cues, which consequently become a powerful source of motivation for future behavior (Flagel et al., 2011). In ST, incentive stimuli become attractive, eliciting approach towards them and promoting potentially maladaptive cue-controlled behaviors; ST individuals, indeed, are generally more vulnerable to addiction and relapse (Tomie et al., 1998; Flagel et al., 2008; Robinson and Flagel, 2009). The ST and GT profiles do not seem to be limited to the CR expressed, but are also associated with differences in traits such as impulsivity; ST individuals are characterized by higher levels of impulsive behavior compared to GT individuals (Tomie et al., 2000; Flagel et al., 2009).

A deeper investigation into individual differences in attributing incentive salience to reward-paired stimuli would thus be important for understanding and reducing the propensity to develop maladaptive behaviors.

The aim of the present study was to investigate individual differences in human PIT. Specifically, the present study explored, for the first time in humans, whether individual differences in the propensity to approach and engage a Sign (cue-predicting reward) or a Goal (reward) are predictive of cue-controlled behavior. To this end, a typical PIT experimental design was used, comprising three tasks. In the first phase, participants performed an Instrumental Conditioning task, in which they were presented with two possible choices, one paired with an actual monetary win (Rewarded Choice) and the other paired with a neutral outcome (Unrewarded Choice). In a subsequent session, participants performed a Pavlovian Conditioning task, during which they learned to associate a specific visual cue with an actual monetary win (CS+), and another visual cue with a neutral outcome (CS−). During this phase, eye-movements were recorded and subsequently analyzed in order to identify the expressed CR and characterize participants as ST or GT. Mirroring previous studies conducted in animals (Boakes, 1977; Flagel et al., 2007, 2008, 2011; Saunders and Robinson, 2013), in which the CR is identified based on the amount of approaching behavior expressed during CS presentation, in the present study ST and GT participants were distinguished based on a learned oculomotor CR. Specifically, it was measured the tendency to direct contiguous eye-gazes toward the location where the visual CS (Sign) or the reward (Goal) would be presented. Finally, PIT was tested in an extinction phase (without any rewards), during which participants had to choose between the same two options given during instrumental conditioning, while presented with the task-irrelevant CS. In this final phase, PIT would be observed if presentation of the CS+, compared to the CS−, enhanced instrumental responses to the choice rewarded during instrumental conditioning (Congruent Choice), relative to the previously unrewarded choice (Incongruent Choice). If consistent with animal literature, this effect should be stronger in ST individuals than in GT individuals, possibly indicating a stronger biasing effect of Pavlovian cues over behavior in the first group relative to the second.

## Method

### Participants

Forty-five volunteers (27 female; 2 left-handed; mean age = 24.87, sd = 2.5; mean education = 17.53, sd = 1.5) with no history of neurological diseases were recruited from the student population at the University of Bologna. All participants gave written informed consent to take part in the experiment and received payment corresponding to the amount earned during the tasks. The study was conducted in accordance with institutional guidelines and the 1964 Declaration of Helsinki. It was approved by the Ethics Committee for Psychological Research at the University of Bologna.

### Stimuli and Procedure

**Figure 1:**
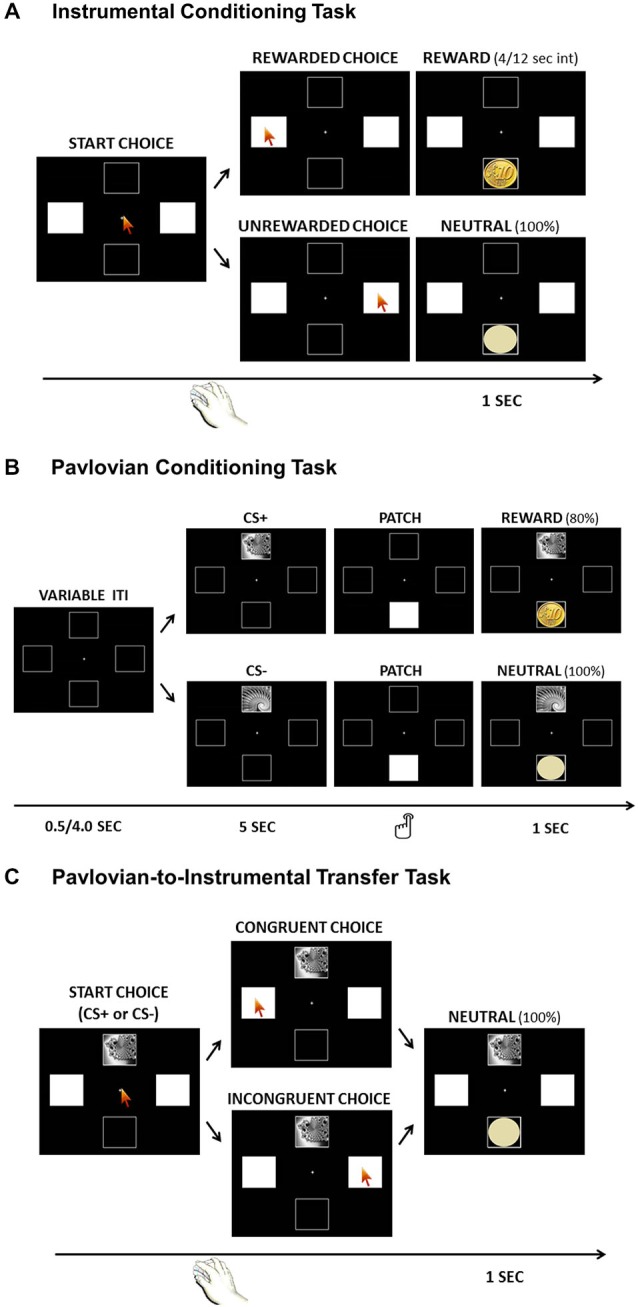
**Graphical illustration of the three tasks: Instrumental Conditioning Task (Panel A), Pavlovian Conditioning Task (Panel B); Pavlovian-to-Instrumental Transfer (PIT) Task (Panel C)**.

The whole experiment consisted of three tasks. The same visual background was used in all three tasks. Four black squares (4 cm^2^) were displayed on a 17-inch color monitor with a black background. The squares were highlighted by a white frame and positioned as follows: top center, bottom center, right center, left center. Two black-and-white fractal images (balanced for luminance, complexity and color saturation) were used as Pavlovian cues (CS) and presented within the top center square. An image of a 10 euro cent coin was used as the reward, and a light-yellow circle (equally sized) was used as the neutral outcome (no-reward). Both these visual cues appeared within the bottom center square (Figure 1). A computer running Presentation software (Neurobehavioral Systems, Albany, CA, USA) controlled stimulus presentation. On arrival, participants were comfortably seated in a silent room and their position was centered relative to the screen, at a viewing distance of 60 cm from the eye-tracker and 75 cm from the screen. The eye-tracker was positioned under the screen, and was centered relative to both the screen and the participant. Eye-movements and behavioral responses were collected throughout the experiment and stored for offline analysis. Participants were asked to remain as still as possible to avoid confounding effects on eye-movements. The whole experiment was conducted in a dark room to facilitate eye-movement recording. The experimental session began with calibration of the eye-tracker device, during which the participant fixated nine specific points on the computer screen. The experimental session followed the standard paradigm for testing PIT. It was composed of three tasks administered in succession: an Instrumental Conditioning task, in which participants learned a response-contingent reward; a Pavlovian Conditioning task, in which participants learned a cue-contingent reward; and a PIT task, during which the influence of irrelevant Pavlovian cues on instrumental responding was tested. In each task, participants were required to pay attention to the screen and follow the instructions reported at the beginning of the task. A few example trials were always performed and, if necessary, further clarifications were given before beginning each task. At the end of the experimental session, participants completed the Barratt Impulsiveness Scale (BIS-11; Patton et al., 1995). Previous studies on animals reported an association between Sign-Tracking behavior and reduced impulse control (Flagel et al., 2011). Thus, this measure allowed further investigation into the differences between ST and GT individuals.

#### Instrumental Conditioning Task

Participants were instructed to choose between two squares to gain a reward. One square was paired with an actual monetary win (Rewarded Choice), while the other was paired with a neutral outcome (Unrewarded Choice). The right and left squares were presented in white and indicated as possible choices to be selected by a mouse click. The mouse pointer was centrally positioned before each choice, in order to not encourage a specific choice. Only one square was associated with a reward following a partial reinforcement schedule, so that between one reward and the next a variable interval between 4 and 12 s was always associated with no-reward. After each choice, a corresponding neutral image (light-yellow circle) or reward image (10 euro cents coin) appeared for 1 s in the bottom square (Figure 1A). Participants were aware that they would receive an actual payment corresponding to the amount of coins collected during the task. The association between square and outcome was counterbalanced across subjects. The rationale of this task was to make participants learn an association between a specific response (left or right square) and the reward; thus, participants would get a higher frequency of Rewarded Choices if they learned the correct association. The task lasted about 6 min, during which subjects were free to perform as many choices as they wished, with no time pressure.

#### Pavlovian Conditioning Task

In each trial, one of two possible visual cues (fractal images) appeared for 5 s within the top square, followed by a white patch within the bottom square. Upon presentation of the patch, participants were instructed to press the left-Ctrl button on the keyboard as quickly as possible to remove the patch and discover the outcome hidden below. To perform this button press, participants did not need to remove their gaze from the screen. The outcome was then presented for 1 s. One fractal was associated with a reward (10 euro cent coin) on 80% of trials (CS+), while the other fractal was associated with no-reward (light-yellow circle) on all trials (CS−; Figure 1B). The task consisted of 40 trials (20 per condition) with a variable inter-trial-interval between 0.5 and 4 s. Participants were aware that they would receive an actual payment corresponding to the amount of coins collected during the task. The association between visual cue and outcome was counterbalanced across subjects. The whole task lasted around 6 min.

The Pavlovian speeded reaction time response described above (“press the button upon patch presentation”) has been successfully used in previous studies Talmi et al. (2008) and was introduced to obtain a behavioral measure of Pavlovian conditioning. The main reason for using a speeded response was to mirror PIT studies on animals, in which Pavlovian conditioning is measured by a behavior performed to gain the reward (e.g., latency of the first nose-poke or frequency of nose-pokes; Dickinson et al., 2000; Holland, 2004; Corbit and Balleine, 2005). The rationale here is to observe a faster reaction times when a reward was predicted (CS+ condition) than when a neutral outcome was predicted (CS− condition). To avoid a possible instrumental influence on the task, participants were explicitly told that, in this task, the reward was not contingent on their response. It was demonstrated that, if no answer was given, the patch would disappear anyway after 1.5 s, revealing the outcome. Importantly, this speeded reaction time response allowed us to obtain a measure of the learning rate that is independent from ST/GT behavior.

To identify ST and GT CR, eye-movements were recorded in order to evaluate contiguous eye-gazes directed toward the “Sign” (top center square) and the “Goal” (bottom center square). Mirroring animal studies, these two CR were subsequently used to distinguish participants as ST or GT, depending on the tendency to direct eye-gaze toward the Sign or the Goal during the 5 s of CS presentation (Flagel et al., 2011).

#### Pavlovian-to-Instrumental Transfer (PIT) Task

Participants received exactly the same instructions as in the Instrumental Conditioning phase requiring them to choose between the right and left white squares. The task was identical to the Instrumental Conditioning task, except in two aspects: first, the task-irrelevant Pavlovian CS were presented sequentially within the top square, changing every 30 s, the task was completely performed in extinction, so all choices always lead to no-reward. (Figure 1C). Extinction is a standard procedure for assessing PIT, both in human and animal research, since it allows one to test the influence of Pavlovian cues on instrumental responding without the confounding effects of the reward (Rescorla, 1994a,b; Corbit et al., 2001; Bray et al., 2008; Talmi et al., 2008). Indeed, the rationale here is to test the ability of a task-irrelevant Pavlovian cue to drive choices (presumably, towards the response previously associated with a reward) even if the reward is not available anymore. The PIT task lasted about 6 min, during which subjects were free to perform as many choices as they wished, with no time pressure.

### Eye Tracking

Eye movements were recorded in a dimly lit room using a Pan/Tilt optic eye-tracker (Eye-Track ASL-6000) which registers real-time gaze at 50 Hz. Data acquired during the Pavlovian Conditioning task were analyzed offline using EyeNal Analysis Software (ASL). Dwell time during the 5 s of CS presentation was then measured for two specific areas of interest (AOI): “Sign”, corresponding to the 4 cm square at the top center, plus a 1 cm margin; “Goal”, corresponding to the 4 cm square at the bottom center, plus a 1 cm margin. Dwell time was defined as the amount of time during which a series of contiguous fixations remained within the same AOI.

### Sign-Tracker and Goal-Tracker Categorization

Participants were categorized as ST or GT based on the oculomotor CR expressed during the Pavlovian Conditioning task. Previous studies used approaching and engaging behaviors during Pavlovian Conditioning to identify ST and GT. In these studies, the numbers of contacts with the Sign (i.e., lever) and the Goal (i.e., food tray) were compared to obtain an index of behavior, and divide the subjects into ST (i.e., high probability to engage the lever) and GT (i.e., high probability to engage the food-tray) individuals (Flagel et al., 2007, 2008, 2011; Robinson and Flagel, 2009; Saunders and Robinson, 2013; Robinson et al., 2014). This method was adapted in the present experiment by calculating contiguous eye-gazes (Dwell Time) toward the cue (Sign) and the reward (Goal) AOI, during CS presentation (see above). ST behavior has been defined as a CR to approach and engage “the cue or sign that indicates impending reward delivery”; while GT behavior has been defined as a tendency to “engage the location of unconditioned cue delivery, even though it is not available until conditioned cue termination” (Flagel et al., 2011). Thus, a learned oculomotor CR towards the location of the Sign or the Goal is a practical method for distinguishing between ST and GT individuals. On this basis, an eye-gaze index was created based on the Dwell Time spent on the Sign and Goal locations. An individual dwell is defined as the time period during which a fixation or series of temporally contiguous fixations remain within an AOI. That is, an individual dwell is defined as the sum of the durations across all fixations within the current AOI, from entry to exit. To compute fixations, EyeNal ASL was used, which defines a fixation if the observer’ s gaze position remains within a diameter of 0, 5° of visual angle for at least 120 ms (six consecutive samples, at 50 Hz sampling rate; Eye-Analysis software Manual, v. 1.41, Applied Science Laboratories, 2007). The Dwell Time spent on the Sign and Goal locations was calculated for each trial and then averaged for each participant. The eye-gaze index was calculated as the difference between the Dwell Time on Sign minus the Dwell Time on Goal over the total Dwell Time (Sign − Goal/Sign + Goal), so that a higher value corresponded to a higher Dwell Time toward the Sign (Sign-Tracking behavior) and a lower value corresponded to a higher Dwell Time toward the Goal (Goal-Tracking behavior). Since the interest here was to disentangle two reward-specific CR, only CS+ trials in the second half of the task were considered, when contingency learning was more established. Based on this index, the top and bottom 50% of the total sample were categorized as ST (eye-gaze index between 0.38 and 1.00) and GT (eye-gaze index between −1.00 and 0.27), respectively.

## Results

### ST and GT CR

**Figure 2:**
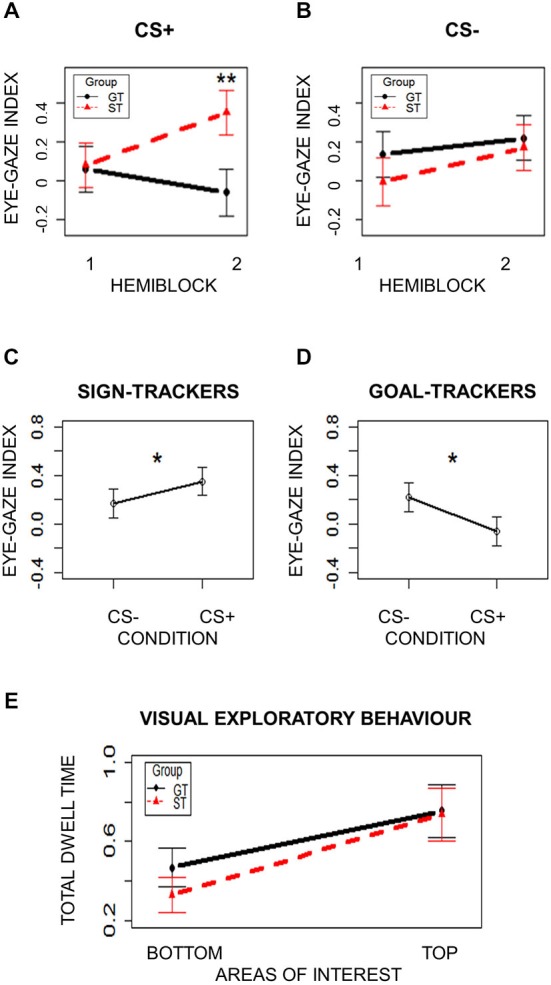
**Oculomotor response**. **(Panels A** and **B)** show the eye-gaze index in the two groups (ST = Sign-Trackers; GT = Goal-Trackers) and the two task hemiblocks. **(Panel A)** represents CS+ trails and **(Panel B)** represents CS− trials. **(Panels C** and **D)** show the eye-gaze index in the two conditions (CS+ = reward-associated cue; CS− = neutral cue) in ST and GT, respectively. **(Panel E)** shows visual exploratory behavior in the two groups (ST = Sign-Trackers; GT = Goal-Trackers) throughout the task. Bars indicate standard error of the mean. *p < 0.05; **p < 0.01.

To ensure that the oculomotor responses used to categorize ST and GT individuals were learned CRs, eye-gaze indices were separately analyzed for CS+ and CS− trials in the first and second halves of the Pavlovian Conditioning task. Two separate mixed-effects models with Group (ST/GT) and Hemiblock (1/2) as independent variables were performed for CS+ and CS− conditions. The eye-gaze index described above was the dependent variable. Subjects were modeled as a random effect. Assumptions of normal distribution, independence of residuals and sphericity were verified. Results from CS+ trials showed a significant interaction effect (*F*_(1,42)_ = 14.75; two-tailed *p* = 0.0004; part. *η*^2^ = 0.26). Bonferroni-corrected *post hoc* tests revealed a significant difference (*p* = 0.003) between ST (mean = 0.35; sd = 0.77) and GT (mean = −0.06; sd = 0.79) in the second Hemiblock (Figure 2A). No other *post hoc* comparisons were significant (*p*s > 0.15). Results from CS− trials did not show any significant effects (*p*s > 0.05; Figure 2B). Overall, these results indicate two important points: first, a bias toward either the Sign or the Goal is a learned CR, since it is not present at the beginning of the task but emerges later in time, when contingencies have been learned (Figure 2A); moreover, this looking bias is specific to the reward-paired cue (CS+), as no differences were observed for the unpaired cue (CS−; Figure 2B). In Figure 2A it is evident how, at the beginning of the Pavlovian task, during CS+ presentation, no tendency seems evident, while, towards the end ST show higher Dwell Time towards the Sign (eye-gaze index increases) while GT show higher Dwell Time towards the Goal (eye-gaze index decreases). Figure 2B, on the other hand, shows that the same pattern is not observable during the presentation of the neutral stimulus (CS−).

To further test that this behavior is a reward-specific CR, the eye gaze index was also directly compared between CS+ and CS− trials from the second hemiblock (when contingencies had been learned) within each group. Two separate paired *t*-tests were performed for the ST and GT groups, using Condition (CS+/CS−) as the independent variable and the eye-gaze index as the dependent variable. In both groups a significant difference between the two conditions was found. The ST group showed a significantly higher eye-gaze index in the CS+ condition than in the CS− condition (*t*_(21)_ = 1.69; one-tailed *p* = 0.03; Cohen’s *d* = 0.19), indicating a greater tendency to direct contiguous eye-gazes towards the Sign during CS+ trials than during CS− trials (Figure 2C). The GT group showed a significantly lower eye-gaze index in the CS+ condition than in the CS− condition (*t*_(21)_ = 2.21; one-tailed *p* = 0.01; Cohen’s *d* = 0.24), indicating a greater tendency to direct contiguous eye-gazes towards the Goal during CS+ trials than during CS− trials (Figure 2D).

Given the specific spatial locations of the Sign and the Goal in the present paradigm, visual exploratory behavior was also considered by analyzing the total dwell time spent on the top and the bottom portions of the screen, in order to exclude the presence of a spatial bias that could account for ST and GT behavior. A mixed-effects model was used, with Group (ST/GT) and AOI (Top/Bottom) as independent variables and Total Dwell Time as dependent variable. Subjects were modeled as a random effect. Assumptions of normal distribution, independence of residuals and sphericity were verified. Results showed a marginal main effect of AOI (*F*_(1,42)_ = 4.01; two-tailed *p* = 0.05; part. *η*^2^ = 0.09), with more Dwell Time spent on the Top of the screen (mean = 0.76; sd = 0.91) than on the Bottom (mean = 0.41; sd = 0.64) in both groups (Figure 2E). Neither group differences, nor interaction effects emerged (*p*s > 0.87). These results strengthen the evidence that the behavioral differences observed between ST and GT cannot be ascribed to a mere spatial bias towards the upper or the lower part of the screen. The general difference in time spent looking at the Top and the Bottom of the screen is compatible with the fact that dwell time was calculated during the 5 s of CS presentation. These results thus indicate that both groups spent more time visually exploring the region of the screen where a stimulus was being presented (Top), rather than where there was no stimulus (Bottom). No difference in this spatial bias was found between the two groups (Figure 2E).

Taken together, the last two analyses demonstrated that group differences in the tendency to direct contiguous eye-gazes to the location of the Sign or the Goal cannot be ascribed to a mere spatial bias, but rather reflect a learned reward-related CR.

### Instrumental Conditioning

**Figure 3:**
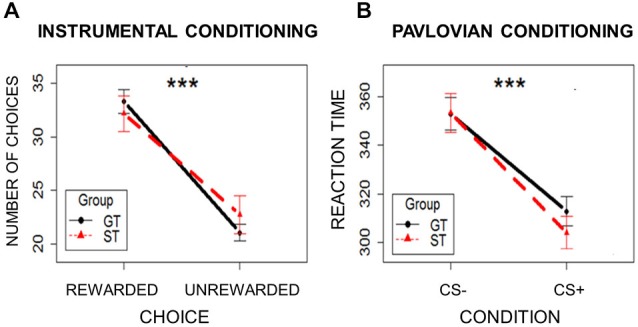
**Learning rates in the two groups (ST = Sign-Trackers; GT = Goal-Trackers) during Instrumental Conditioning (Panel A) and Pavlovian Conditioning (Panel B)**. Bars indicate standard error of the mean. ***p < 0.001.

To ensure that instrumental conditioning was successful in both the ST and the GT groups, so that all participants learned which response leads to a reward, the number of choices (mouse clicks) made on the two white squares were compared. Choosing the square associated with reward was considered a Rewarded Choice, and choosing the square associated with no-reward was considered an Unrewarded Choice. A mixed-effects model was used, with Choice (Rewarded/Unrewarded) and Group (ST/GT) as independent variables and the number of choices as the dependent variable. Subjects were modeled as a random effect. Assumptions of normal distribution, independence of residuals and sphericity were verified. Results showed a main effect of Choice (*F*_(1,42)_ = 20.88; two-tailed *p* < 0.0001; part *η*^2^ = 0.33), with Rewarded Choices (mean = 32.80; sd = 9.38) occurring more frequently than Unrewarded Choices (mean = 22.09; sd = 9.10; Figure 3A). Neither group differences, nor interaction effects emerged (*p*s > 0.55). These results indicate that the ST and GT groups learned to discriminate between the rewarding and non-rewarding choices equally well.

### Pavlovian Conditioning

To ensure that Pavlovian learning occurred in both ST and GT groups, reaction times to patch presentation were analyzed. If participants correctly learned to discriminate between the two Pavlovian cues, faster reaction times should be observed for CS+ trials relative to CS− trials. A mixed-effects model was used, with Condition (CS+/CS−) and Group (ST/GT) as independent variables, and reaction times as the dependent variable. Subjects were modeled as a random effect. Assumptions of normal distribution, independence of residuals and sphericity were verified. Results showed a significant main effect of Condition (*F*_(1,842)_ = 110.24; two-tailed *p* = 0.0001; part. *η*^2^ = 0.72), with faster reaction times for CS+ trials (mean = 306.33; sd = 44.41) relative to CS− trials (mean = 351.21; sd = 50.05; Figure 3B). Neither group differences, nor interaction effects emerged (*p*s > 0.29). These results indicate that participants generally reacted more quickly to the patch on trials with the reward-paired cue (CS+) than on trials with the unpaired cue (CS−). This reward-specific response facilitation indicates successful Pavlovian conditioning in both ST and GT.

### Pavlovian-to Instrumental Transfer

**Figure 4:**
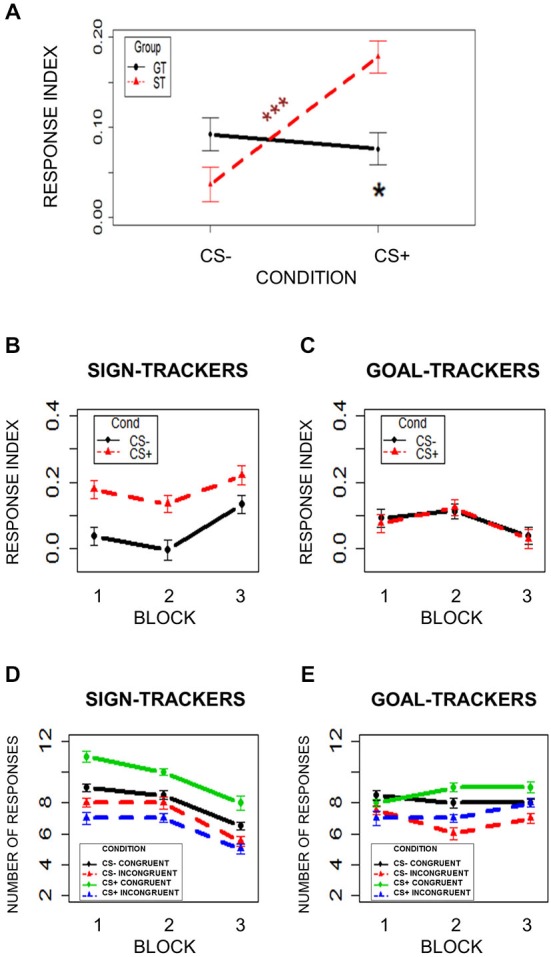
**Pavlovian-to-Instrumental Transfer (PIT)**. **(Panel A)** shows the response index (Congruent-Incongruent/Total) in the two groups (ST = Sign-Trackers; GT = Goal-Trackers) during CS− and CS+ trials. **(Panels B** and **C)** show the response index over time by dividing the task into three blocks of two trials. **(Panels D** and **E)** show the number of responses. Bars indicate standard error of the mean. *p < 0.05; ***p < 0.001.

To test for PIT, the numbers of Congruent choices (associated with the reward during Instrumental Conditioning) and Incongruent choices (associated with no-reward during Instrumental Conditioning) during CS+ and CS− presentation were compared. A response index was calculated as the probability of selecting the Congruent choice minus the probability of selecting the Incongruent choice (number of congruent—incongruent choices/total number of choices). Higher values correspond to a higher probability of making the Congruent choice, while lower values correspond to a higher probability of making the Incongruent choice. A mixed-effects model was used, with Condition (CS+/CS−) and Group (ST/GT) as independent variables and the response index, described above, as the dependent variable. Subjects were modeled as a random effect. Assumptions of normal distribution, independence of residuals and sphericity were verified. Results showed a significant Condition × Group interaction (*F*_(1,42)_ = 8.22; two-tailed *p* = 0.006; part. *η*^2^ = 0.16). Bonferroni-corrected *post hoc* comparisons revealed a significant difference (*p* = 0.001) between CS+ (mean = 0.18; sd = 0.12) and CS− (mean = 0.04; sd = 0.13) only in ST group, and a significant difference (*p* = 0.04) between ST (mean = 0.18; sd = 0.12) and GT (mean = 0.08; sd = 0.12) during CS+ (Figure 4A). No other comparisons were significant (*p*s > 0.13). These results indicate that the ST group was more likely to choose the congruent option when they saw the task-irrelevant CS+ than when they saw the CS−. thus revealing a PIT effect. Critically, this bias was stronger in ST than in GT individuals.

While the first analysis on PIT focused on the overall effect, a second analysis divided the task into three equal blocks of 2 min (four trials) to check for differences in task performance over time. A mixed-effects model was used, with Condition (CS+/CS−), Group (ST/GT) and Block (1/2/3) as independent variables, and the response index as the dependent variable. Subjects were modeled as a random effect. Assumptions of normal distribution, independence of residuals and sphericity were verified. Results showed a significant main effect of Condition (*F*_(1,42)_ = 6.39; two-tailed *p* = 0.02; part. *η*^2^ = 0.13), a significant Condition × Group interaction (*F*_(1,42)_ = 7.69; two-tailed *p* = 0.008; part. *η*^2^ = 0.15), and a significant Block × Group interaction (*F*_(1.27,53.32)_ = 50.61; two-tailed *p* < 0.001; part. *η*^2^ = 0.5; Figures 4B,C). Bonferroni-corrected *post hoc* tests on the Condition × Group interaction revealed a significant difference (*p* = 0.003) between CS+ and CS− in ST group but not the GT group, and a significant difference (*p* = 0.02) between ST and GT groups in CS+ trials (Figures 4B,C). Bonferroni-corrected *post hoc* tests on the Block × Group interaction revealed a significant difference (*p* < 0.0001) between ST and GT groups in the third block, but not in the first and second blocks (Figures 4B,C). Figures 4D,E show the number of responses.

In line with the results of the first analysis, these results showed that, unlike GT, ST group was more likely to choose the congruent option when they saw the task-irrelevant CS+ than when they saw the CS−, throughout the entire PIT task. The only effect of time revealed by this analysis was in the last block, where a group difference in responses emerged. Since this difference was unrelated to the displayed stimulus (CS+/CS−), it does not constitute a difference in PIT. This result instead indicates that the ST and GT groups differed in the proportion of congruent choice made towards the end of the task.

### Impulsiveness

**Figure 5:**
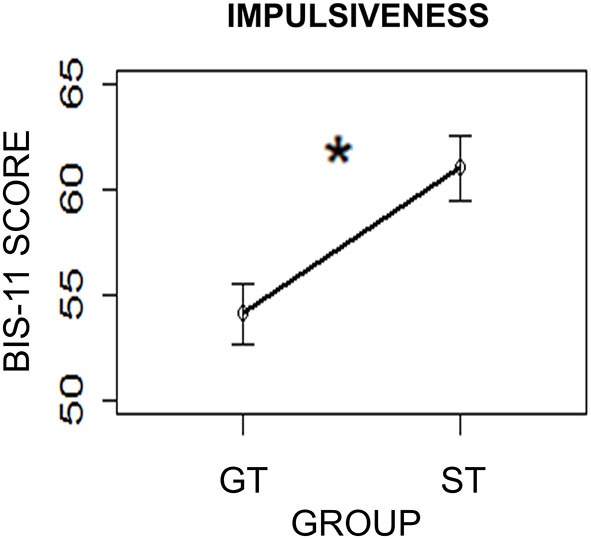
**Impulsiveness levels in the two groups (ST = Sign-Trackers; GT = Goal-Trackers) as measured by the Barratt Impulsiveness Scale (BIS-11)**. Bars indicate standard error of the mean. *p < 0.05.

To further investigate differences between ST and GT individuals, self-reported impulsiveness, as rated by the BIS-11 questionnaire (Patton et al., 1995), was compared between the two groups. A two-sample *t*-test was performed using Group (ST/GT) as the independent variable and BIS-11 scores as the dependent variable. Results revealed a significant difference between the two groups (*t*_(28.75)_ = 2.06; two-sided *p* = 0.04, with the ST group (mean = 61.0; sd = 9.91) showing higher impulsiveness than the GT group (mean = 54.09; sd = 8.86; Figure 5). This finding is consistent with previous studies showing significantly higher levels of impulsiveness as compared to GT (Tomie et al., 2000; Flagel et al., 2009).

## Discussion

Motivated behavior is characterized by a wide span of inter-individual differences in both human and non-human animals. In the present study, the PIT paradigm was used to examine individual differences in the excitatory influence that signals associated with reward can exert on human choices. PIT is a well-known procedure for testing the ability of a Pavlovian reward-paired cue to acquire incentive motivational properties and influence instrumental performance (Estes, 1943, 1948; Rescorla and Solomon, 1967; de Wit and Dickinson, 2009; Holmes et al., 2010). Here, participants performed a standard PIT paradigm composed of three tasks: an Instrumental Conditioning task, during which response-outcome associations were learned; a Pavlovian Conditioning task, during which stimulus-outcome associations were leaned; and a PIT task, in which the ability of a Pavlovian cue to drive instrumental responses was tested. Individual differences were characterized by two distinct oculomotor CR exhibited during Pavlovian Conditioning, corresponding to two different learning styles previously identified and described in animal literature: Sign-Tracking (ST) and Goal-Tracking (GT; Estes, 1943, 1948; Boakes, 1977; Flagel et al., 2011). In the present study, ST behavior consisted of a tendency to direct contiguous eye-gazes towards the cue (CS) that indicated impending reward delivery (Sign); in contrast, GT behavior was characterized by a tendency to direct contiguous eye-gazes towards the location of reward (US) delivery (Goal), even if not available until CS termination. An eye-gaze index was based on the emergence of these two behavioral patterns during presentation of the reward-paired stimulus (CS+) in the second half of the task (when contingencies had been learned), and a median split was used to categorize participants as ST or GT. Importantly, the present results demonstrate that this oculomotor CR was: (i) acquired over time (i.e., learned), since a specific CR towards the Sign or the Goal only emerged towards the end of the task, when stimulus-reward associations had been acquired selectively during the presentation of reward-paired cues (CS+; Figures 2A,B); and (ii) reward specific, since the CR was only evident when participants saw the reward-related cue (CS+) and not when they saw the neutral cue (CS−; Figures 2C,D). Coherently with what expected, the task-irrelevant CS had a much stronger influence on the ST group than on the GT group during the PIT task.

Group differences in the PIT effect are not attributable to differences in the strength of Instrumental or Pavlovian learning between the groups, which could have potentially induced a bias towards the rewarded choice in the Instrumental Conditioning task, or a stronger influence of the reward-paired cue in the second Pavlovian Conditioning task. Analyses of both the number of rewarded choices during Instrumental Conditioning, and reaction times during Pavlovian Conditioning, exclude such a possibility by revealing that both the ST and GT groups learned the response-outcome and stimulus-outcome contingencies equally well (Figure 3). Consequently, differences in the PIT effect cannot be explained by group differences in the ability to learn either the instrumental or the Pavlovian contingencies. In line with the animal literature (Robinson and Flagel, 2009), the Pavlovian cue (CS+) was clearly predictive of reward, since it elicited faster reaction times during Pavlovian conditioning than the neutral stimulus (CS−) did in both groups, along with a CR corresponding to the behavioral profile of each group (ST/GT).

Since the “Sign” and the “Goal” had specific spatial locations (the top and bottom portions of the screen, respectively), it is important to rule out the possibility that spatial biases in gaze direction might account for the difference in the PIT effect between groups. A bias towards looking at the top of the screen might cause result in a stronger influence of the Sign on the ST group just because they spent more time looking at it. Analysis of visual exploratory behavior during Pavlovian Conditioning, however, revealed that the ST and GT groups did not differ in the total amount of time spent looking at the top and bottom of the screen (Figure 2E). Critically, behavioral differences only emerged during CS+ trials towards the end of the task, once the association between the cue and the reward had been learned. Consequently, it is concluded that there was no *a priori* bias in gaze direction; rather, such a bias emerged during the Pavlovian Conditioning task as a learned reward-specific CR.

Moreover, a recent study (Trick et al., 2011) directly investigated the relation between fixation times during Pavlovian learning and the PIT effect. The authors found that fixation times during Pavlovian learning increased with uncertainty (that is, more attention was paid to stimuli with uncertain outcome probabilities, e.g., 50%, than to stimuli with more certain outcome probabilities, e.g., 90%). In contrast, the PIT effect increased with the probability of reward (that is, it was stronger for stimuli associated with a high probability of reward, e.g., 90%, than for stimuli associated with uncertain outcomes, e.g., 50%, or a low probability of reward, e.g., 10%). Thus, Trick et al. (2011) concluded that the behavioral influence exerted by CS (i.e., the PIT effect) is dissociated from attention to Pavlovian stimuli in humans, (see Kaye and Pearce, 1984, for similar findings in animals). Instead, PIT is linked to the predictive value acquired by stimuli during learning.

ST behavior has been explained as a consequence of attributing incentive salience to reward-paired cues (Pavlovian CS), arising from the interaction between previous experience (reinforcement learning processes) and individual propensities (Berridge, 2001; Berridge and Robinson, 2003; Flagel et al., 2011). This incentive salience motivates reward-related action (Tomie et al., 2000; Flagel et al., 2008; Robinson and Flagel, 2009). In the present study, ST and GT groups differed in the extent to which Pavlovian reward-paired cues biased their behavior: relative to the GT group, the ST group showed an increased likelihood of performing the instrumental response independently paired with the same reward when presented with the task-irrelevant reward-paired cue, even if the reward itself was no longer available (i.e., a stronger PIT effect; Figure 4A). Therefore, reward-paired cues exerted a stronger source of influence on the behavior of ST individuals, as predicted. Importantly, time course analysis revealed that this effect occurred early and remained stable throughout the entire PIT test session (Figures 4B,C), thereby suggesting that the group difference in the PIT effect most likely reflects greater incentive salience to reward cues in ST than in GT individuals. A group difference in the overall amount of congruent responses (during both CS+ and CS− presentation, thus not reflecting PIT) emerged towards the end of the task (Figures 4B,C).

Previous studies have found an association between ST behavior and other traits, such as higher levels of behavioral impulsivity and a greater propensity to develop addiction (Tomie et al., 1998; Flagel et al., 2008; Robinson and Flagel, 2009). In line with these studies, the present study found reduced self-reported impulse control in the ST group than in the GT group (Figure 5). These findings seem to corroborate the idea that ST and GT behaviors are just one expression of a broader profile of individual differences, which might be clinically relevant. Many studies have reported that ST individuals are more impulsive and prone to develop potentially maladaptive behaviors, such as addiction (Tomie et al., 1998; Robinson and Flagel, 2009; Flagel et al., 2011). For example, the propensity to sign-track is associated with a stronger effect of psychomotor sensitization, a higher susceptibility to a form of cocaine-induced plasticity that may contribute to the development of addiction (Flagel et al., 2008). Furthermore, ST behavior in relation to a specific Pavlovian cue (i.e., a cue predicting monetary reward) is also predictive of the propensity to attribute incentive salience to other reward-paired cues, such as food-related or drug-related cues (e.g., cocaine and alcohol; Uslaner et al., 2006; Cunningham and Patel, 2007; Flagel et al., 2008; Clark et al., 2013). The extent to which such individual differences might play a role in the development of addiction and in the propensity to relapse is not yet clear, but their implications for developing individually targeted treatment programs are promising.

It should be noted that some recent studies highlighted a more complex scenario relating ST and GT behaviors to addiction. While ST individuals are more susceptible to the influence of discrete cues, GT individuals are more influenced by contextual cues, which can motivate drug-seeking behavior (Robinson et al., 2014). Consequently, these learning styles seem to reflect differences in the kinds of triggers to which the individual is susceptible (e.g., discrete/contextual), rather than a propensity to addiction *per se*. This finding emphasizes that there are diverse pathways to addiction, and has remarkable implications for the development of personalized treatments in the future.

But what exactly is the mechanism underlying the attribution of incentive salience to discrete stimuli, such as Pavlovian cues? A large amount of evidence points to the role of dopaminergic transmission within circuits known to be involved in addiction. The core of the nucleus accumbens, for example, was reported to be involved in ST behavior, and mediates the reinstatement of drug-seeking and drug-taking behavior (Flagel et al., 2007, 2008, 2011; Clark et al., 2013). Furthermore, various studies have supported the involvement of the mesolimbic dopamine system in the emergence of ST behavior. ST individuals are characterized by stronger dopaminergic gene expression and increased levels of dopamine in the nucleus accumbens (correlated with the vigor with which the CR is performed; Flagel et al., 2007, 2008). Even if differences in basic dopaminergic levels cannot fully account for differences in dopamine responsiveness, it has been argued that higher reward-related dopamine release before conditioning might increase attribution of incentive salience to reward-related cues (Wyvell and Berridge, 2000, 2001). Additionally, Flagel et al. (2011) directly demonstrated that dopaminergic transmission is not involved in all forms of learning, but it is necessary for the acquisition of a sign-tracking CR, playing a crucial role in the assignment of incentive salience to reward-related cues. The same study also showed that dopaminergic prediction-error signals, coded by activity in the nucleus accumbens, are present in ST individuals, but not in GT individuals. In the present study, a similar mechanism might occur: high levels of dopamine release might boost attribution of incentive salience to reward-related cues, increasing their ability to motivate and drive behavior.

Future studies might further investigate individual differences in the influence of Pavlovian cues on behavior by taking additional measures into account, such as phasic dopamine levels, psychophysiological indices (e.g., galvanic skin response and heart rate) and as neuroimaging measurements. These methods would allow better comparisons between human and non-human animal research on individual differences in ST/GT behavior and learning styles. A general limitation in the standard PIT paradigm is that the “Sign” and the “Goal” are presented in distinct spatial locations. Thus, unrelated spatial biases in gaze direction might obscure the effect of interest. Although the analysis conducted in this study already confirmed that the present findings cannot be accounted for by any *a priori* difference in spatial bias between groups, another way to control for this possibility would be to replicate the experiment with the spatial positions of the “Sign” and the “Goal” inverted in the three tasks.

In conclusion, the individual differences demonstrated here offer a promising direction for further investigating the degree to which incentive salience is attributed to environmental stimuli associated with rewards, as well as the link between this process and maladaptive behaviors, ranging from over-eating to pathological gambling and addiction (Saunders and Robinson, 2013). Further, the present findings have important implications for the treatment of impulse-control disorders. Overall, these individual differences in PIT offer new insights into the mechanisms underlying the transition from intentional to habitual/compulsive behavior.

## Author Contributions

All authors conceived of and designed the experiment; S. G. programmed the task, ran the experiment, analyzed the data, wrote the main manuscript text and prepared the figures; all authors read, corrected and approved the final manuscript.

## Conflict of Interest Statement

The authors declare that the research was conducted in the absence of any commercial or financial relationships that could be construed as a potential conflict of interest.
